# Application of Natural Pigments in Ordinary Cooked Ham

**DOI:** 10.3390/molecules25092241

**Published:** 2020-05-10

**Authors:** Sandra Dias, Elisabete M. S. Castanheira, A. Gil Fortes, David M. Pereira, A. Rita O. Rodrigues, Regina Pereira, M. Sameiro T. Gonçalves

**Affiliations:** 1 Centre of Chemistry, Department of Chemistry, University of Minho, Campus of Gualtar, 4710-057 Braga, Portugal; sandraisdias@hotmail.com (S.D.); gilf@quimica.uminho.pt (A.G.F.); 2 Centre of Physics, Department of Physics, University of Minho, Campus of Gualtar, 4710-057 Braga, Portugal; ecoutinho@fisica.uminho.pt (E.M.S.C.); ritarodrigues@fisica.uminho.pt (A.R.O.R.); 3 REQUIMTE/LAQV, Laboratory of Pharmacognosy, Department of Chemistry, Faculty of Pharmacy, University of Porto, R. Jorge Viterbo Ferreira, 228, 4050-313 Porto, Portugal; dpereira@ff.up.pt; 4 Primor Charcutaria-Prima, S.A, Avenida Santiago de Gavião, n° 1142 Gavião, 4760-003 Vila Nova de Famalicão, Portugal; regina.pereira@primor.pt

**Keywords:** ordinary cooked ham, beetroot, natural dyes, betalain pigments, betacyanins

## Abstract

The possibility of obtaining a carmine or pink color on ordinary cooked ham by applying natural dyes from three plant species, namely red radish (*Raphanus sativus* L.), hibiscus (*Roselle sabdariffa* L.) and red beetroot (*Beta vulgaris* L.), was investigated. The extracts were evaluated for the stability at physical-chemical parameters and subjected to cytotoxicity assays in the gastric cell line AGS Encapsulation of the extracts in soybean lecithin liposomes and maltodextrin microcapsules was performed. Lyophilized extracts before and after encapsulation in maltodextrin were applied in the formulation of ordinary cooked ham and used in a pilot scale of production. The color of cooked ham samples from different assays was evaluated visually and by colorimetry. The results suggest that the coloration of ordinary cooked ham obtained with extracts of red beetroot is very promising for future applications in this type of meat product.

## 1. Introduction

Processed meat includes products ranging from those with a minimum of 30% meat, to products that include 100% meat. The preparation of meats for added value products can be considered the definition of processing. This can comprise portioning, forming and processing procedures, for example emulsification, salting, curing, marinating, cooking, smoking or drying [1]. Concerning the quality assessment and process optimization, it is imperative that the quality of the ingredients used, the production process itself and the final products are carefully monitored. This includes the evaluation of several parameters in the meat used, during the production process, as well as of the final product, making color assessment part of all stages of process monitoring. 

According to Portuguese Standard 4393 (NP 4393) of 2001, cooked ham is defined as a meat product prepared exclusively from pork, brine, whether it is pressed, and subsequently subjected to heat treatment [2]. This standard classifies cooked ham in five categories: (1) upper leg cooked ham, (2) extra leg cooked ham, (3) leg cooked ham, (4) shoulder arm cooked ham, and (5) ordinary cooked ham, according to the cut of pork and the ingredients that can be used in its preparation. In the case of ordinary cooked ham, the type of cooked ham that was the focus of the present work, it is a product prepared from pork leg meat to which the ingredients mentioned in NP 4393 are added, namely the following essential ingredients: meat, drinking water and ice, refined salt; and optional ingredients: sugars, flavors, meat and non-meat proteins, starches, additives in accordance with the legislation and the provisions of the standard, and topping jellies. In this type of ham, the minimum amount of protein is 12%. The optional ingredients could be important in the physicochemical and sensory characteristics of the final product, which must be presented in the form of a block or slices and the cut must have a moist surface with characteristic smell and taste [3]. 

Processed meats are expected to retain the cherry red color of fresh meat. Natural ingredients and biological agents used in meat processing are safe and very well tolerated, thus representing an excellent opportunity to highlight a certain characteristic, for example the color, without harmful effects. 

Among the natural pigments that have been used to improve the color of many foods and beverages are anthocyanins, such as pelargonidin-3-*O*-diglucoside-5-*O*-glucoside and its acylated forms with residues of caffeic, β-coumaric, ferulic, and/or malonic acid present in the rhizomes of red radish (*Raphanus sativus*) from the *Brassiceae* family [4,5,6,7,8,9,10]. The stability of anthocyanins depends on several parameters, such as pH, temperature, and oxidative conditions, but they are normally quite stable in acidic media [11,12,13]. Since they are polyphenolic compounds, they also possess strong health-promoting effects as antioxidants [14,15]. With a similar composition to the red radish, hibiscus (*Roselle sabdariffa* L.) of the *Malvaceae* family also contains cyanidin, cyanidin 3-sambubioside, and delphinidin 3-*O*-sambubioside, which contribute to the antioxidant activity of this plant [16,17,18,19,20,21,22]. Betalain pigments such as betacyanins (red-violet color) and betaxanthins (yellow-orange color), along with a variety of other components with numerous nutritional and health benefits, are found in red beetroot (*Beta vulgaris* L.) from the *Amaranthaceae* family [23,24,25]. The pigments contain predominantly two betacyanins (isobetanine and betanine) and three betaxanthins (indicaxanthin, vulgaxanthin I, and vulgaxanthin II) [24,26,27], which give to beetroot its characteristic color and contribute to its antioxidant quality [25,26]. The use of beetroot as an ingredient in several products imparts beneficial effects on human health and provides an opportunity for development of different functional foods [28,29,30].

Considering all these facts, the present work investigated the possibility of obtaining a carmine/pink color in ordinary cooked ham by applying natural dyes from the three plant species mentioned above, namely red radish, red beetroot, and hibiscus. Extraction procedures were performed in order to obtain the respective extracts containing natural dyes, which were analyzed by UV-Vis and evaluated for stability at different physical-chemical parameters (pH, temperature and light). The cytotoxicity of various extracts was evaluated in the human gastric adenocarcinoma cell line AGS, frequently employed to mimic gastric cells [31,32]. Nanoencapsulation of extracts was performed in liposomes of soybean lecithin and maltodextrin capsules. The extracts obtained from the aforementioned species after lyophilization and incorporation in maltodextrin were applied in the formulation of ordinary cooked ham and used in its pilot scale. The color of ham samples from the different assays was evaluated visually and by colorimetry. The results suggest that ordinary cooked ham coloration obtained with lyophilized extracts of red beetroot, is very promising for future applications in this type of meat product.

## 2. Results and Discussion

### 2.1. Anthocyanin- and Betalain-Rich Extracts

**Figure 1:**
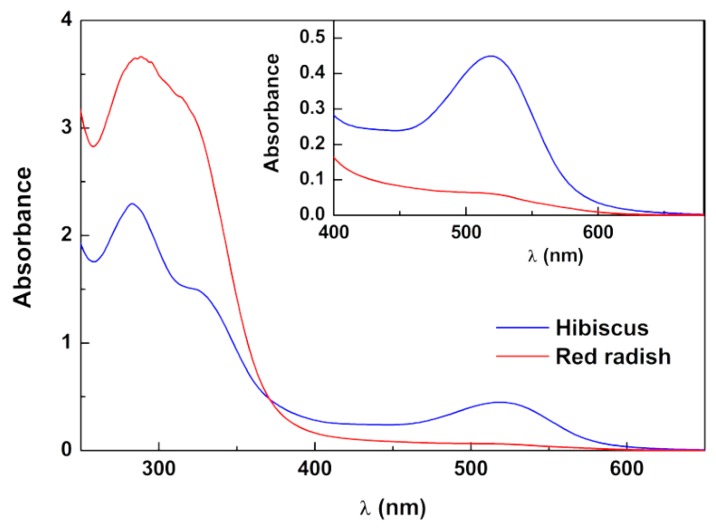
Absorption spectra of aqueous solutions of lyophilized extracts of red radish (concentration: 1.47 × 10^−3^ g/mL) and hibiscus (concentration: 1.73 × 10^−3^ g/mL). Inset: Expansion of the spectra in the visible region.

With the aim of obtaining anthocyanin-rich extracts from red radish and hibiscus, different experimental conditions, including various solvent systems, were used. Acid aqueous solutions, specifically water/acetic acid (95:5, *v*/*v*) (red radish) and water/ethanol/acetic acid (70:29.7:0.3, *v*/*v*/*v*) (hibiscus), at low temperature (−4 °C), with irradiation protection for 18 h (red radish) and 72 h (hibiscus), resulted in high absorbance liquid extracts at about 507 or 531–542 nm, respectively, suggesting significant concentrations of anthocyanin pigments [33]. Consequently, these extracts were subjected to lyophilization. The UV-Vis absorption spectrum of an aqueous solution of lyophilized extract of red radish, with a broad band in the visible region with absorption maxima at 507 nm, is compatible with the presence of anthocyanin pigments (Figure 1) [5,9]. Similarly, hibiscus extract displayed maximum absorption wavelength at 519 nm (Figure 1), which is in accordance with literature regarding anthocyanins absorption present in hibiscus [34,35]. Considering the UV-Vis data and applying Equation (1)

anthocyanins content (mg/L) = (A × DF × MW × 1000)/ε × *l* (1)

where A is the maximum absorbance at 512 nm (red radish) and 519 nm (hibiscus), DF is the dilution factor, and *l* is the optical path (1 cm), the anthocyanin contents were estimated, resulting in 1.27 g/L and 13.01 g/L for red radish and hibiscus, respectively.

**Figure 2:**
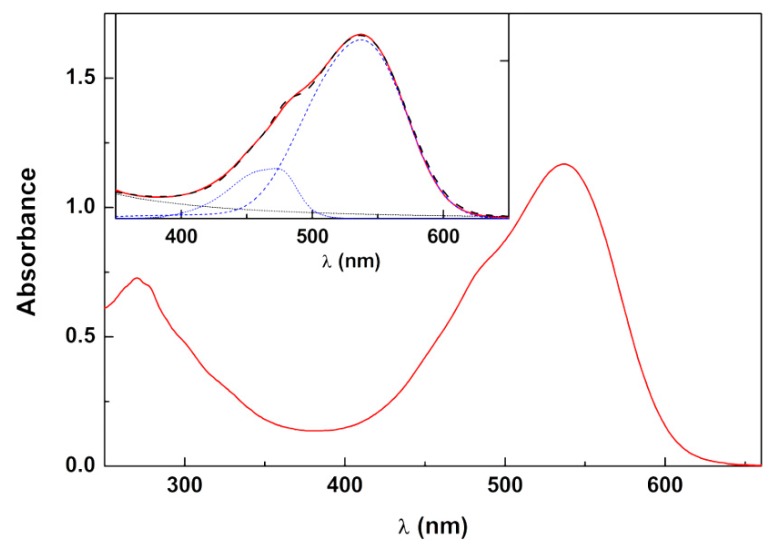
Absorption spectrum of red beetroot lyophilized extract in aqueous solution (at a concentration of 1.73 × 10^−3^ g/mL). Inset: Spectral decomposition in the visible region to obtain betacyanins and betaxanthins content.

For the purpose of obtaining betalain-rich extracts from red beetroot, a similar procedure to that described in obtaining the anthocyanin extracts was followed. After testing several experimental conditions, the use of acidic aqueous-ethanoic media, namely a water/ethanol/acetic acid (66.6:33:0.33, *v*/*v*/*v*) solution as solvent system, at room temperature, with irradiation protection for 48 h, resulted in high absorbance liquid extract at about 538 nm and 480 nm, which discharged the presence of anthocyanins and suggested the presence of significant concentrations of betalain pigments. Therefore, this extract was subjected to lyophilization. The UV-Vis absorption spectrum of the extract of red beetroot showed a broad band with absorption maxima at 537 nm and a shoulder at 480 nm, suggesting the presence of betacyanins and betaxanthins, respectively (Figure 2). Thus, the spectrum in the visible region was decomposed (inset of Figure 2) in the betacyanins and betaxanthins components to obtain an estimation of the betalains content, using the reported spectra for each of the components [36]. The decomposition (using a fitted baseline) of the experimental absorption spectrum is shown in the inset of Figure 2. This procedure allows an estimation of 20.1 mg/L of betacyanins and 4.27 mg/L of betaxanthins (after compensation of the dilution factor).

**Figure 3:**
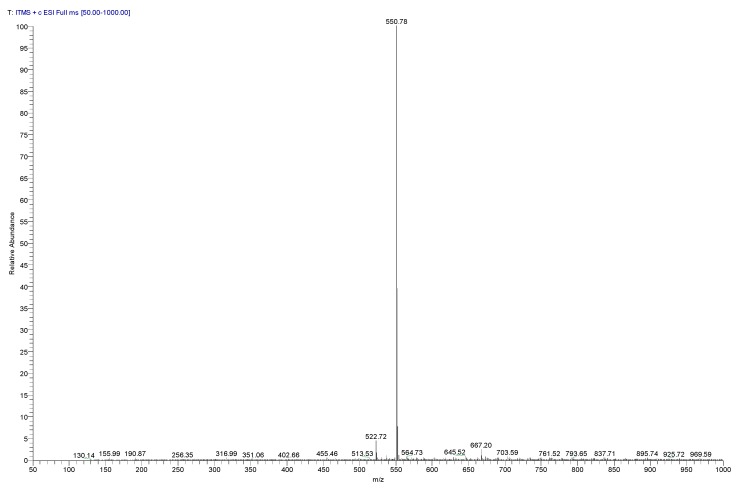
Low resolution mass spectrum acquired in positive ionization mode (ESI) of red beetroot lyophilized extract with peaks at 550.78 (100.00 %) and 551.78 m/z (39.50%).

Low resolution mass spectrometry acquired in positive ionization mode (ESI) displayed a peak with m/z 550.78 (base peak) attributed to betanin, according to the literature related to red beetroot pigment components [25] (Figure 3).

**Figure 4:**
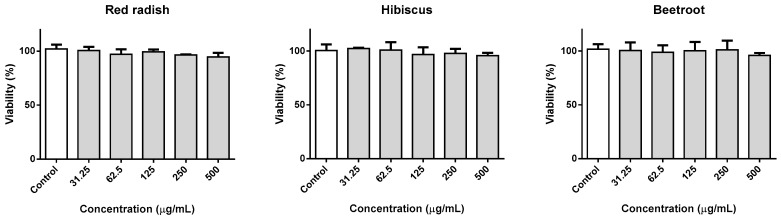
Viability of AGS cells exposed to red radish, hibiscus, and red beetroot extracts for 24 h, in the 31.25–500 µg/mL range.

Given the specific cooked ham application intended for this work, as well as other potential food applications, the cytotoxicity of lyophilized extracts of red radish, hibiscus and red beetroot in human gastric adenocarcinoma (AGS) cells, as a model for gastric cells, was evaluated. The extracts were evaluated for their potential effect upon gastric cells, following 24 h of exposure. Even at the highest concentration tested, 0.5 mg/mL, no loss of viability was detected (Figure 4), which points to the lack of toxicity of all extracts.

**Figure 5:**
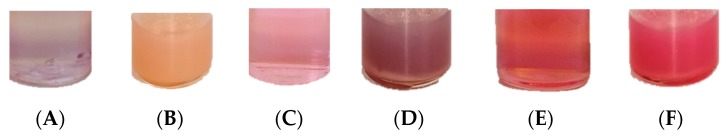
Lyophilized extracts in water (**A**), (**C**), (**E**) and brine (**B**), (**D**), (**F**) of red radish (**A**), (**B**), hibiscus (**C)**, (**D**), and red beetroot (**E**), (**F**).

Considering the evaluation of the potential of extracts to give a rosy color to ordinary cooked ham and that the process of it starts with the preparation of the brine, where the extracts are added, preliminary tests were carried out. Thus, each lyophilized extract was added to the brine obtained directly from Primor Charcutaria-Prima, S.A. with the composition to be used in subsequent pilot scale tests, with the extracts in aqueous solutions for comparison (Figure 5).

By visual comparison, extracts in the brine have a different color from that observed only in water as a result of the presence of different components in the mixture. The pink color obtained with the extract of red beetroot is the closest to the desired color. Furthermore, the extracts in brine solutions were also subjected to pH and temperature variations, including heating to 75 °C (the temperature used in the cooking phase of the ordinary cooked ham production), and exposure to light (data not shown), confirming that the red beetroot extract appeared to be the most promising. 

### 2.2. Application of the Extracts to Ordinary Cooked Ham

Since anthocyanins and betalains are sensitive to certain conditions (pH, temperature, and light, among others), and considering their use in coloring of the ordinary cooked ham, it was decided to nanoencapsulate the red radish, hibiscus, and red beetroot extracts. Therefore, liposomes of soybean lecithin and maltodextrin (DE20) matrices were used, as these systems are reported for nanoencapsulation stabilization of different components, as well as additives in the food industry [37]. 

For soybean lecithin, ethanolic injection and thin film hydration methods were used in the preparation of liposomes, as they are usually associated with high encapsulation efficiencies, which, however, may depend on the hydrophilic/hydrophobic nature of the extracts [38,39]. The results obtained (Table 1) showed high encapsulation efficiencies for the three extracts, without a substantial difference between the two preparation procedures.

However, since the industrial scale application of these processes has both financial and experimental limitations, incorporation in maltodextrin (DE20) capsules was also carried out, which is easier to perform and economically favorable. The results showed a ~100% yield of encapsulation. Consequently, later, maltodextrin encapsulation was used in the pilot scale tests.

The main objective of this work was to evaluate the possibility of obtaining a rosy color in ordinary cooked ham by using natural dyes that, in addition to color, can imparts beneficial effects on human health. Hence, extracts of red radish, hibiscus, and red beetroot, non-encapsulated and encapsulated in maltodextrin after lyophilization, and the commercial carmine dye (E120, C.I. 75470), used for comparison, were added to the formulation and the corresponding ordinary cooked ham preparations were made on a pilot scale at Primor Charcutaria-Prima, S.A. (Vila Nova de Famalicão, Portugal). The E120 from the cochineal insect was used for comparison, since it is capable of giving the carmine/pink color, is available on the market, and is already used in the food industry [40,41,42,43]. Although carmine and its derivatives present some benefits in comparison with artificial and other natural colorants, several drawbacks, such as health issues and social refusal, have shifted the food industry to consider other natural alternatives [44,45]. 

For evaluation of the results obtained in the ordinary cooked ham with the tested extracts, it was used in the ordinary cooked ham produced and marketed by Primor Charcutaria-Prima, S.A., and the ordinary cooked ham prepared by adding E120 with a concentration of 0.1 g/kg (concentration achieved by a preliminary study with variable concentrations of dye; data not shown). These two ordinary cooked hams represent, respectively, the currently existing color that is to be improved and the optimum color that is intended to be obtained with natural pigments. 

**Figure 6:**
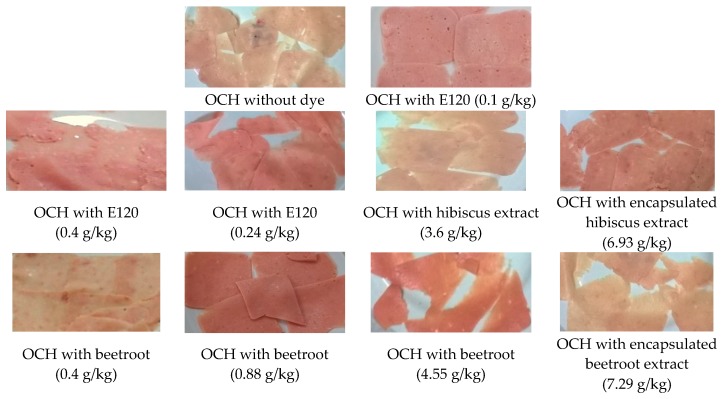
Samples in slices from the pilot scale ordinary cooked ham (OCH) obtained without dye, with E120, and the non-encapsulated and encapsulated extracts of hibiscus and red beetroot with various concentrations.

Figure 6 presents the results obtained in the ordinary cooked ham, without dye, by applying the E120 and the lyophilized extracts of hibiscus and red beetroot with the extract mass/kg of cooked ham applied in the different tests.

**Figure 7:**
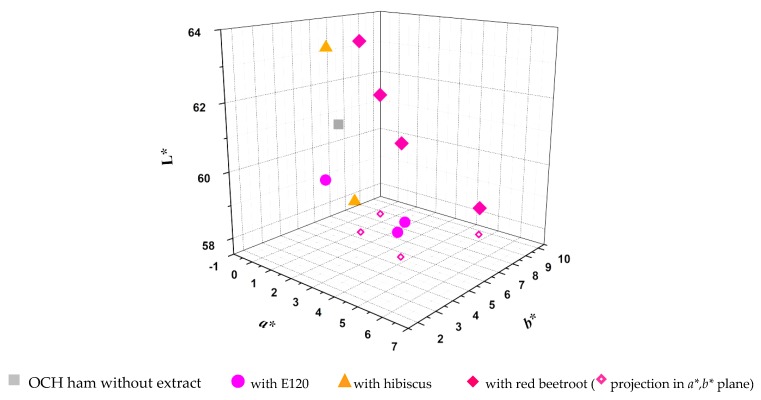
Representation of the colors of the several samples in the L*a*b* color scheme.

The color obtained in ordinary cooked ham samples was evaluated visually and by measuring color indexes in a colorimeter, where L* is the degree of lightness and covers a range from black (0) to white (100), index a* is the degree of redness and greenness (from −80 to 0 = green; from 0 to 100 = red), and b* is the degree of yellowness and blueness (from −100 to 0 = blue; from 0 to +70 = yellow) (Table 2). For a better visualization, the L*a*b* color representation is shown in Figure 7.

In the case of red radish extract, the desired color for ordinary cooked ham was far from being achieved, both in the application of the direct extract or incorporated in maltodextrin (data not shown). It was observed that the color of the ordinary cooked ham samples was dark in both cases. The experiments with hibiscus were performed using non-encapsulated and encapsulated extracts and bearing very close pigment concentrations. The samples of ordinary cooked ham obtained show different index a*, the result being closer to the expected (a* = 1.84) due to the application of the encapsulated extract (a* = 2.19). Regarding the index b*, the encapsulated extract (b* = 4.79) shows the next desired value (b* = 3.59). Considering the general color evaluation by the colorimeter, together with the final visual aspect of the ordinary cooked ham slices where the hibiscus extract was applied, the best result is given by the extract incorporated in maltodextrin. 

The experiments with red beetroot were carried out with the non-encapsulated extract with different concentrations, and extract incorporated in maltodextrin. The results show different a* values, the result closest to those obtained with E120 (0.1 g/kg) (a* = 1.84) being achieved by the application of red beetroot extract with a concentration of 0.4 g/kg (a* = 1.06). Regarding the b* values, the non-encapsulated extract with a concentration of 0.88 g/kg presents a value (b* = 5.66) near to the desired one (b* = 3.59). The lightness parameter L* is close in both experiments with encapsulated and non-encapsulated extracts. The results of the color parameters corresponding to the experiments with red beetroot extracts, together with the final visual appearance of the four ordinary cooked ham samples, suggest that the best result occurred in the application of the non-encapsulated extract with concentrations of 0.4 and 0.88 g of extract/kg of ordinary cooked ham, which, when projected on the *xy*-plane (color plane a*, b*; Figure 6) is clearly close to the ones of samples with E120 with 0.24 and 0.4 g/kg of ordinary cooked ham.

## 3. Experimental Section

### 3.1. Chemicals and Reagents

Ethanol, isopropanol, DMSO, acetic acid and tetrahydrofuran were purchased from Merck KGaA (Darmstadt, Germany). Trypan blue and 3-(4,5-dimethylthiazolyl-2)-2,5-diphenyltetrazolium bromide (MTT) were from Sigma-Aldrich (St. Louis, MO, USA). Dulbecco’s Modified Eagle Medium (DMEM), Hank’s balanced salt solution (HBSS), foetal bovine serum (FBS), penicillin-streptomycin solution (penicillin 5000 units/mL and streptomycin 5000 μg/mL), and 0.25% trypsin-EDTA were obtained from GIBCO, Invitrogen™ (Grand Island, NY, USA). The dye E120 was a Mane Iberica S.A. product (Barcelona, Spain). Ultrapure water was Milli-Q grade (MilliporeSigma, St. Louis, MO, USA).

### 3.2. Plant Material

The red radish, hibiscus and red beetroot were obtained from a local grocery store in Portugal as fresh materials (red radish and red beetroot) or in dehydrated form (hibiscus) in October 2019. The parts of the plants used were: in the case of red radish only the peels; in the case of the red beetroot only the interior after being peeled. The hibiscus was used directly in the form in which it was purchased.

### 3.3. Preparation of Anthocyanin-Rich Extracts 

To the red radish peel cut into small pieces (25 g), a solution of water/acetic acid (95:5, *v*/*v*) (100 mL) was added, and the mixture was kept at low temperature (~4 °C), for 18 h. After this time, the mixture was filtered, and the solvent was partially evaporated in the rotary evaporator (Rotavapor^®^ R-210, BÜCHI Labortechnik AG, Flawil, Switzerland) at 40 °C. Then, the extract was frozen at −80 °C and lyophilized (Alpha 1–4 LD Plus–Christ freeze dryer, Martin Christ Gefriertrocknungsanlagen GmbH, Osterode am Harz, Germany) for five days, resulting in the corresponding dry extract that was stored in a desiccator until use. 

To the hibiscus (5 g), a solution of water/ethanol/acetic acid (70:29.7:0.3 *v*/*v*/*v*) (10 mL) was added, and the mixture was kept at low temperature (~4 °C), for 72 h, and a similar procedure as the one described above for red radish was followed.

### 3.4. Preparation of Betalain-Rich Extracts

To the red beetroot, previously hand-peeled and cut into small pieces (5 g), a solution of water/ethanol/acetic acid (66.6:33:0.33, *v*/*v*/*v*) (10 mL) was added, and the mixtures were kept at room temperature, for 48 h. After this time, the mixture was filtered and the solvent partially evaporated in the rotary evaporator (Rotavapor^®^ R-210, BÜCHI Labortechnik AG, Flawil, Switzerland) at 40 °C. Then, the extracts were frozen at −80 °C and lyophilized (Alpha 1–4 LD Plus – Christ freeze dryer, Martin Christ Gefriertrocknungsanlagen GmbH, Osterode am Harz, Germany) for five days, resulting in the corresponding dry extract that was stored in a desiccator until ready to be used.

### 3.5. Determination of Anthocyanins Content

The absorbance values of the extracts of red radish and hibiscus were measured at 512 and 519 nm, respectively, against a blank of ultrapure water, on a Shimadzu 3600 Plus UV-Vis-NIR spectrophotometer (Shimadzu Corporation, Kyoto, Japan). These values were then used to calculate the anthocyanins content in the extracts of red radish and hibiscus using Equation (1). Molecular weight (MW) and molar absorptivity (ε) of pelargonidin-3-*O*-glucoside from red radish (MW = 433.2 g/mol; ε = 3.16 × 10^4^ M^−1^ cm^−1^) and cyanidin-3-*O*-sambubioside from hibiscus (MW = 449.2 g/mol; *ε* = 2.69 × 10^4^ M^−1^ cm^−1^) were used [33,34]. 

### 3.6. Determination of Betalains Content

The absorption spectrum of red beetroot extract were fitted to a sum of betacyanins spectrum and betaxanthins spectrum to obtain the content of each dye, which are reported as mg equivalent betanin/L and mg equivalent indicaxanthin/L, respectively. Betacyanins were detected at 538 nm and betaxanthins at 480 nm. For betacyanin, *ε* = 6.5 × 10^4^ M^−1^ cm^−1^ and MW = 550 g/mol, while for betaxanthins, *ε* = 4.8 × 10^4^ M^−1^ cm^−1^ and MW = 308 g/mol [46,47]. 

### 3.7. LRMS Analysis of Extracts

LRMS analyses of red beetroot lyophilized extract were carried out on a Thermo Scientific™ LTQ XL™ linear ion trap mass spectrometer (Thermo Fisher Scientific, MA, USA) [25]. 

### 3.8. AGS Cell Assays

As a model of toxicity after oral consumption, the human gastric adenocarcinoma cell line AGS was used. Cells were purchased from Sigma-Aldrich (St. Louis, MO, USA) and maintained in medium DMEM +GlutaMAxTM-1 with 1% penicillin/streptomycin and 10% FBS, at 37 °C, in a humidified atmosphere of 5% CO_2_. 

For the assessment of viability, cells were plated at a density of 1.5 × 10^4^ cells/well, followed by incubation for 24 h with the samples. After incubation, 0.5 mg/mL MTT solution was added and further incubated for 2 h. The formazan in each well was dissolved in a solution of DMSO/isopropanol (3:1). Lastly, the absorbance at 560 nm was read in a Thermo Scientific™ Multiskan™ GO microplate reader (Massachusetts, MA, USA).

### 3.9. Nanoencapsulation Studies

For nanoencapsulation studies, the lyophilized extracts of radish, hibiscus, and beetroot were used. Concentration dilutions of 2 × 10^−5^–5 × 10^−3^ mg/mL were performed to determine the calibration curve and calculate the encapsulation efficiency.

Liposomes were prepared by the ethanolic injection and thin film hydration methods, using a commercial lipid mixture for the food industry, soybean lecithin (Sternchemie, Hamburg, Germany), containing (%mol/mol) 22% phosphatidylcholine, 20% phosphatidylethanolamine, 20% phosphatidylinositol and 10% phosphatidic acid as main components, with a concentration of 1 × 10^−3^ M. In the ethanolic injection method, 2 × 10^−3^ g/mL extract was added to the lipid mixture and the solvent was evaporated with an ultrapure nitrogen stream. After evaporation, tetrahydrofuran (0.75 µL) and ethanol (0.75 µL) were added. Under vortexing, the mixture was added to ultrapure water (3 mL) and the resulting solution was placed in Amicon tubes and centrifuged (Universal 320 Hettich Zentrifugen, New Delhi, India) for 10 min at 3000 rpm.

For thin film hydration, soybean lecithin (60 × 10^−3^ g) was added to ethanol (3 mL) and the mixture was evaporated under a stream of ultrapure nitrogen. To the lipid mixture, 10 × 10^−3^ g/mL of the aqueous extract solution, previously dissolved in 5 mL of ultrapure water, was added. This solution was vortexed for 5 min and sonicated also for 5 min. Then, the resulting mixture was centrifuged in Amicon filter tubes for 10 min at 3000 rpm.

Both the encapsulated and non-encapsulated fractions were collected, and absorption spectra were recorded in a Shimadzu UV-3600 Plus UV-Vis-NIR spectrophotometer (Shimadzu Corporation, Kyoto, Japan). The absorbance of the fractions was measured and the concentrations of the encapsulated and non-encapsulated pigments were determined, using the calibration curve (absorbance vs. concentration). The encapsulation efficiency, EE (%), was obtained through the relation: EE (%) = (Total amount-Amount of non-encapsulated extract) / (Total amount) × 100. 

For the assays using maltodextrin (DE20), the lyophilized extract (0.5 g) was added to distilled water (3 mL) and then, while stirring, maltodextrin (3 g) was added. The resulting mixture was centrifuged (Hermle LaborTechnik GmbH, Wehingen, Germany) at 500 rpm for 16 min. and then subjected to lyophilisation and, based on the final mass obtained, the encapsulation yield EY (%) was calculated by: EY (%) = (final mass after lyophilisation) / (initial mass (maltodextrin + extract)) × 100 [48].

### 3.10. Application of Red Radish, Hibiscus and Red Beetroot Extracts to Ordinary Cooked Ham

The ingredients that make up the brine and the different meats included in the ordinary cooked ham were weighed. The brine was prepared and E120, the extracts of red radish, hibiscus or red beetroot were added. The temperature and pH of the resulting mixture were measured. The meat was later added and, with the help of a food processor (Yämmi SPM-018, Sonae SA, Porto, Portugal), the mixture was blended to make it homogeneous. The resulting mixture was then packaged in plastic casing, formed into metal containers, and kept for 12 h at 4 °C.

After resting, the paste was cooked at 75 °C, which is achieved when the core reaches 72 °C. At this point, the ordinary cooked ham was quickly placed on ice to rapidly reach 4 °C and stored at low temperatures (5 °C) for 24 h. At the end of this period, the ordinary cooked ham was deformed and placed at a low temperature of 4 °C until used for colorimetric assays.

### 3.11. Color Measurement

The CIELAB parameters, L* (brightness), a* (green vs. red coordinate) and b* (blue vs. yellow coordinate), were determined on a white calibration block using a Chroma Meter CR-400/410 colorimeter (Konica Minolta, Tokyo, Japan). 

The ordinary cooked ham samples, without and with E120, red radish, hibiscus or red beetroot extracts in the formulation used in their preparation, were cut into slices of about 1.5 mm in thickness. The color measurements were made in a transparent Petri dish (5 cm^2^), where the ham slices were placed to be analyzed. Each sample was analyzed at five distinct points.

### 3.12. Statistical Analysis

For biological assays, a Shapiro–Wilk normality test was performed on the data to ensure that it followed a normal distribution. Comparison between the means of controls and each experimental condition was performed using ANOVA. Outliers were identified by Grubbs’s test. Data was expressed as the mean ± standard error of the mean of three independent experiments, each performed in triplicate. GraphPad Prism software was used and values were considered statistically significant with a *p* ≤ 0.05.

## 4. Conclusions

Anthocyanin-rich extracts of red radish and hibiscus, as well as betalain-rich extracts of red beetroot were evaluated for their stability at different physical-chemical parameters, and subjected to cytotoxicity assays in gastric cells, which revealed the absence of toxicity. Incorporation of the extracts in nanosystems based on soybean lecithin and maltodextrin resulted in high encapsulation efficiencies for the three species tested.

Lyophilized extracts of red radish, hibiscus and red beetroot, before and after incorporation in maltodextrin were applied in the formulation of ordinary cooked ham and used in a pilot scale of production. The color of ordinary cooked ham containing E120 dye (0.1 g of dye/kg of ordinary cooked ham) was used as comparison. Visual evaluation, together with the values of color parameters (obtained in colorimeter) for all the ordinary cooked ham samples, suggested that the extract from red beetroot (0.88 g of extract/kg of ordinary cooked ham) was the one that provides the intended color. The results obtained with encapsulated extract of hibiscus also presented color indexes close to the intended one, but the amount of extract required (3.66 g/kg) was much higher than the one from red beetroot.

Overall, the results obtained in this work suggest that the ordinary cooked ham coloration achieved with lyophilized extracts of red beetroot is very promising for future applications in this type of processed meat product.

## Figures and Tables

**Table 1:** Encapsulation efficiency, EE (%) ± SD (%), of extracts in liposomes of soybean lecithin prepared by the two methods (SD: standard deviation).

Species	Ethanolic Injection	Thin Film Hydration
Red radish	99.9 ± 6.2	99.6 ± 4.2
Hibiscus	99.2 ± 4.8	98.1 ± 2.9
Red Beetroot	98.4 ± 3.1	99.3 ± 3.6

**Table 2:** Colorimetric data (L*, brightness; a*, green vs. red coordinate; and b*, blue vs. yellow coordinate) of ordinary cooked ham (OCH) samples prepared without dye and with E120, and extracts of hibiscus and red beetroot.

Sample	Color Parameters		
	L *	a*	b*
OCH without extract	60.74	−0.32	6.84
OCH with E120 (0.1 g/kg)	59.89	1.84	3.59
OCH with E120 (0.24 g/kg)	59.38	5.34	3.59
OCH with E120 (0.4 g/kg)	59.57	6.05	2.22
OCH with hibiscus (3.6 g/kg)	63.22	−0.19	6.03
OCH with encapsulated hibiscus extract (6.93 g/kg) ^1^	59.09	2.19	4.79
OCH with red beetroot (0.4 g/kg)	63.47	1.06	6.52
OCH with red beetroot (0.88 g/kg)	60.91	3.68	5.66
OCH with red beetroot (4.55 g/kg)	58.37	4.64	9.62
OCH with encapsulated red beetroot extract (7.29 g/kg) ^2^	61.54	0.36	8.71

^1^ Mass of the encapsulated extract corresponds to the ratio 3.66 g of non-encapsulated extract/kg. ^2^ Mass of the encapsulated extract corresponds to the ratio 1 g of non-encapsulated extract/kg.

## References

[1] O’Farrell M., Kerry J.P., Kerry J.F. (2011). Online quality assessment of processed meats. Processed Meats, Improving Safety, Nutrition and Quality.

[2] (2002). Cooked Ham and Cooked Shoulder Definition and Characteristics, Norma Portuguesa NP 4393 2001, CT 35 (DGFCQA). https://lojanormas.ipq.pt/product/np-4393-2001/.

[3] Freixanet L. Aditivos e Ingredientes en la Fabricación de Productos Cárnicos Cocidos de Músculo Entero. http://es.metalquimia.com/upload/document/article-es-12.pdf.

[4] Brouillard R., Chassaing S., Isorez G., Kueny-Stotz M., Figueiredo P. (2010). The visible flavonoids or anthocyanins: From research to applications. Rec. Adv. Polyphen. Res..

[5] Giusti M.M., Rodríguez-Saona L.E., Wrolstad R.E. (1999). Molar absorptivity and color characteristics of acylated and non-acylated pelargonidin-based anthocyanins. J. Agric. Food Chem..

[6] Carmo C.S., Nunes A.N., Serra A.T., Ferreira-Dias S., Nogueira I., Duarte C.M.M. (2015). A way to prepare a liposoluble natural pink colorant. Green Chem..

[7] Giusti M.M., Wrolstad R.E. (1996). Characterization of red radish anthocyanins. J. Food Sci..

[8] Giusti M.M., Ghanadan H., Wrolstad R.E. (1998). Elucidation of the structure and conformation of red radish (*Raphanus sativus*) anthocyanins using one- and two-dimensional nuclear magnetic resonance techniques. J. Agric. Food Chem..

[9] Liu Y., Murakami N., Wang L., Zhang S. (2008). Preparative high-performance liquid chromatography for the purification of natural acylated anthocyanins from red radish (*Raphanus sativus* L.). J. Chrom. Sci..

[10] Tamura S., Tsuji K., Yongzhen P., Ohnishi-Kameyama M., Murakami N. (2010). Six new acylated anthocyanins from red radish (*Raphanus sativus*). Chem. Pharm. Bull..

[11] Delgado-Vargas F., Jiménez A.R., Paredes-López O. (2010). Natural pigments: Carotenoids, anthocyanins, and betalains-characteristics, biosynthesis, processing, and stability. Food Sci. Nutr..

[12] Schiozer A.L., Barata L.E.S. (2007). Stability of natural pigments and dyes. Fitos.

[13] Patras A., Bruton N.P., O’Donnell C., Tiwari B.K. (2010). Effect of thermal processing on anthocyanin stability in food; mechanisms and kinetics of degradation. Food Sci. Technol..

[14] Khoo H.E., Azlan A., Tang S.T., Lim S.M. (2017). Anthocyanidins and anthocyanins: Colored pigments as food, pharmaceutical ingredients, and the potential health benefits. Food Nutr. Res..

[15] Bueno J.M., Sáez-Plaza P., Ramos-Escudero F., Jiménez A.M., Fett R., Asuero A.G. (2012). Analysis and antioxidant capacity of anthocyanin pigments. part II: Chemical structure, color, and intake of anthocyanins. Crit. Rev. Anal. Chem..

[16] Cissé M., Bohuon P., Sambe F., Kane C., Sakho M., Dornier M. (2012). Aqueous extraction of anthocyanin from Hibiscus sabdariffa: Experimental kinetics and modeling. J. Food Eng..

[17] Sato K., Goda Y., Yoshihira K., Noguchi H. (1991). Structure and contents of main coloring constituents in the Calyces of Hibiscus sabdariffa and commercial Roselle Color. J. Food Hyg. Soc..

[18] Grajeda-Iglesias C., Figueroa-Espinoza M.C., Barouh N., Baréa B., Fernandes A., Freitas V., Salas E. (2016). Isolation and characterization of anthocyanins from Hibiscus sabdariffa flowers. J. Nat. Prod..

[19] Domínguez-López A., Remondetto G.E., Navarro-Galindo S. (2008). Thermal kinetic degradation of anthocyanins in a roselle (*Hibiscus sabdariffa* L. cv. ‘Criollo’) infusion. J. Food Sci. Technol..

[20] Selim K.A., Khalil K.E., Abdel-Bary M.S., Abdel-Azeim N.A. (2008). Extraction, encapsulation and utilization of red pigments from Roselle (Hibiscus sabdariffa L.) as natural food colorants. AJFS.

[21] Abou-Arab A.A., Abu-Salem F.M., Abou-Arab E.A. (2011). Physico-chemical properties of natural pigments (anthocyanin) extracted from Roselle calyces (*Hibiscus subdariffa*). J. Am. Sci..

[22] Prenesti E., Berto S., Daniele P.G., Toso S. (2007). Antioxidant power quantification of decoction and cold infusions of *Hibiscus sabdariffa* flowers. Food Chem..

[23] Sawicki T., Bączek N., Wiczkowski W. (2016). Betalain profile, content and antioxidant capacity of red beetroot dependent on the genotype and root part. J. Funct. Foods.

[24] Nemzer B., Pietrzkowski Z., Spórna A., Stalica P., Thresher W., Michalowski T., Wybraniec S. (2011). Betalainic and nutritional profiles of pigment-enriched red beet root (*Beta vulgaris* L.) dried extracts. Food Chem..

[25] Chhikara N., Kushwaha K., Sharma P., Gat Y., Panghal A. (2019). Bioactive compounds of beetroot and utilization in food processing industry: A critical review. Food Chem..

[26] Georgiev V.G., Weber J., Kneschke E., Denev P.N., Bley T., Pavlov A.I. (2010). Antioxidant activity and phenolic content of betalain extracts from intact plants and hairy root cultures of the red beetroot *Beta vulgaris* cv. Detroit Dark Red. Plant Foods Hum. Nutr..

[27] Kujala T.S., Vienola M.S., Klika K., Loponen J.M., Pihlaja K. (2002). Betalain and phenolic compositions of four beetroot (*Beta vulgaris* ) cultivars. Eur. Food Res.. Technol..

[28] Ritz T., Werchan C.A., Kroll J.L., Rosenfield D. (2019). Beetroot juice supplementation for the prevention of cold symptoms associated with stress: A proof-of-concept study. Physiol. Behav..

[29] Silva D.V.T., Baião D.S., Silva F.O., Alves G., Perrone D., Aguila E.M.D., Paschoalin V.M.F. (2019). Betanin, a natural food additive: Stability, bioavailability, antioxidant and preservative ability assessments. Molecules.

[30] Oliveira G.V., Morgado M., Pierucci A.P., Alvares T.S. (2016). A single dose of a beetroot-based nutritional gel improves endothelial function in the elderly with cardiovascular risk factors. J. Funct. Foods.

[31] Hall A.J., Tripp M., Howell T., Darland G., Bland J.S., Babish J.G. (2006). Gastric mucosal cell model for estimating relative gastrointestinal toxicity of non-steroidal anti-inflammatory drugs. Prostaglandins Leukot Essent Fatty Acids.

[32] Basque J.-R., Chénard M., Chailler P., Ménard D. (2001). Gastric cancer cell lines as models to study human digestive functions. J. Cell Biochem..

[33] Wentian C., Eric K., Jingyang Y., Shuqin X., Biao F., Xiaoming Z. (2016). Improving red radish anthocyanin yield and off flavour removal by acidified aqueous organic based medium. RSC Adv..

[34] Tavakolifar F., Givianrad M.H., Saber-Tehrani M. (2016). Extraction of anthocyanins from *hibiscus sabdariffa* and assessment of its antioxidant properties in extra virgin olive oil. Fres. Environ. Bull..

[35] Chumsri P., Sirichote A., Itharat A. (2008). Studies on the optimum conditions for the extraction and concentration of roselle (*Hibiscus sabdariffa Linn.*) extract. J. Sci. Technol..

[36] Gonçalves L.C.P., Trassi M.A.S., Lopes N.B., Dörr F.A., Santos M.T., Baader M.J., Oliveira V.X., Bastos E.L. (2012). A comparative study of the purification of betanin. Food Chem..

[37] List G.R., Ahmad M.U., Xu X. (2015). Soybean lecithin: Food, industrial uses, and other applications. Polar Lipids: Biology, Chemistry, and Technology.

[38] Zhang H. (2017). Thin-film hydration followed by extrusion method for liposome preparation. Methods Mol. Biol..

[39] Jaafar-Maalej C., Diab R., Andrieu V., Elaissari A., Fessi H. (2010). Ethanol injection method for hydrophilic and lipophilic drug-loaded liposome preparation. J. Liposome Res..

[40] Sigurdson G.T., Tang P., Giusti M.M. (2017). Natural colorants: Food colorants from natural sources. Annu. Rev. Food Sci. Technol..

[41] 41 Rodriguez-Amaya D.B. (2016). Natural food pigments and colorants. Curr. Opin. Food Sci..

[42] Dapson R.W. (2007). The history, chemistry and modes of action of carmine and related dyes. Biotech. Histochem..

[43] (2015). EFSA Panel on Food Additives and Nutrient Sources added to Food (ANS), Scientific opinion on the re-evaluation of cochineal, carminic acid, carmines (E 120) as a food additive. EFSA J..

[44] Borges M.E., Tejera R.L., Díaz L., Esparza P., Ibañez E. (2012). Natural dyes extraction from cochineal (*Dactylopius coccus*): New extraction methods. Food Chem..

[45] Fernández-López J.A., Angosto J.M., Giménez P.J., León G.L. (2013). Thermal stability of selected natural red extracts used as food colorants. Plant Foods Hum. Nutr..

[46] Stintzing F.C., Herbach K.M., Mosshammer M.R., Carle R., Yi W., Sellappan S., Akoh C.C., Bunch R., Felker P. (2005). Color, betalain pattern, and antioxidant properties of cactus pear (*Opuntia stricta* spp.) clones. J. Agric. Food Chem..

[47] Silva C., Bolanho B.C. (2018). Ultrasonic-assisted extraction of betalains from red beet (*Beta vulgaris* L.). J. Food Process. Eng..

[48] Otálora M.C., Carriazo J.G., Iturriaga L., Nazareno M.A., Osorio C. (2015). Microencapsulation of betalains obtained from cactus fruit (*Opuntia ficus-indica*) by spray drying using cactus cladode mucilageand maltodextrin as encapsulating agents. Food Chem..

